# Anti-Inflammatory Polyketides from an Alga-Derived Fungus *Aspergillus ochraceopetaliformis* SCSIO 41020

**DOI:** 10.3390/md20050295

**Published:** 2022-04-27

**Authors:** Chunmei Chen, Xue Ren, Huaming Tao, Wenteng Cai, Yuchi Chen, Xiaowei Luo, Peng Guo, Yonghong Liu

**Affiliations:** 1 CAS Key Laboratory of Tropical Marine Bio-Resources and Ecology/Guangdong Key Laboratory of Marine Materia Medica, South China Sea Institute of Oceanology, Chinese Academy of Sciences, Guangzhou 510301, China; chenchunmei18@mails.ucas.ac.cn; 2 University of Chinese Academy of Sciences, Beijing 100049, China; 3 Capital Institute of Pediatrics, Beijing 100020, China; rlxue0529@163.com; 4 Guangdong Provincial Key Laboratory of Chinese Medicine Pharmaceutics, School of Traditional Chinese Medicine, Southern Medical University, Guangzhou 510515, China; taohm@smu.edu.cn (H.T.); cwt0825@163.com (W.C.); cyc11136456@163.com (Y.C.); 5 Institute of Marine Drugs, Guangxi University of Chinese Medicine, Nanning 530200, China

**Keywords:** alga-derived fungus, *Aspergillus ochraceopetaliformis*, polyketide, anti-inflammatory activity

## Abstract

A new linear polyketide, named aspormisin A (**1**), together with five known polyketides (**2**–**6**), were isolated from the alga-derived fungus *Aspergillus ochraceopetaliformis* SCSIO 41020. Their structures were elucidated through a detailed comprehensive spectroscopic analysis, as well as a comparison with the literature. An anti-inflammatory evaluation showed that compounds **2**, **5**, and **6** possessed inhibitory activity against the excessive production of nitric oxide (NO) and pro-inflammatory cytokines in LPS-treated RAW 264.7 macrophages in a dose-dependent manner without cytotoxicity. Further studies revealed that compound **2** was active in blocking the release of pro-inflammatory cytokines (IL-6, MCP-1, and TNF-α) induced by LPS both in vivo and in vitro. Our findings provide a basis for the further development of linear polyketides as promising anti-inflammatory agents.

## 1. Introduction

An acute inflammation response is usually triggered by infection or tissue injury, and the subsequent response is mainly mediated by tissue-resident macrophages and mast cells [1]. A variety of pro-inflammatory factors are massively induced and delivered to the site of inflammation afterwards, including vasoactive amines, chemokines, eicosanoids, cytokines, and products of proteolytic cascades; thus, a “cytokines storm” occurs [2].

IL-6, together with TNF-*α* and IL-1, have long been regarded as pro-inflammatory cytokines induced by LPS and have been determined as markers for the systemic activation of pro-inflammatory cytokines [3]. It should be noted that a cytokine storm is a complicated regulation network. IL-6, for example, as a potent inducer of the acute-phase protein response [4], has both pro- and anti-inflammatory properties [5]. On the one hand, IL-6 can attenuate the synthesis of pro-inflammatory cytokines, such as IL-1 and TNF [6,7], without affecting the synthesis of IL-10 and transforming growth factor-*β* (TGF-*β*) among the anti-inflammatory cytokines group. On the other hand, IL-6 inhibits the production of pro-inflammatory cytokines, such as GM-CSF and IFN-*γ* [3]. Moreover, research has shown that the absence of TNF upgraded the morphology and quantity of damaged mitochondria and rapidly switched to an M2 phenotype [8]. Attention should be attached to these indications that cytokines play a dominant role in the regulation of inflammation, and targeting these inflammatory factors is a promising strategy to block the progression of inflammation.

Alga-associated microorganisms have attracted considerable attention and are proven to be a vital source for new compounds with interesting biological activities [9]. The genus *Aspergillus*, one of the main genera of marine-derived fungi, is rich in species [10,11], and marine-derived *Aspergillus* spp. produce structurally diverse secondary metabolites, including fatty acids, polyketides, alkaloids, and others, with a range of bioactivities [12,13].

**Figure 1:**
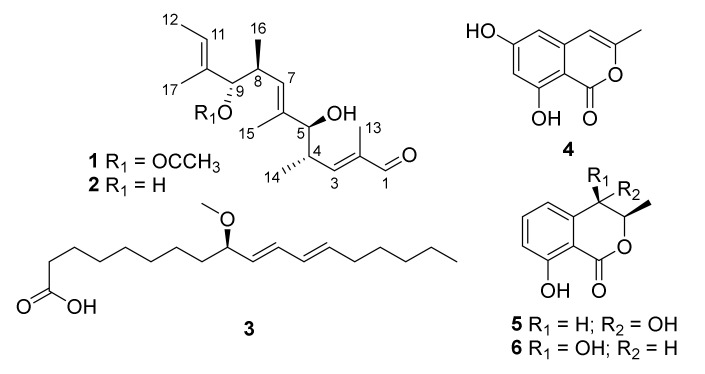
Structures of compounds **1**–**6**.

During our ongoing search for bioactive secondary metabolites from marine-derived fungi [14,15], the alga-derived fungus *Aspergillus ochraceopetaliformis* SCSIO 41020 was selected for investigation due to an interesting HPLC-UV profile of its extract. Six polyketide compounds, including a new linear one, aspormisin A (**1**), were obtained, and several of them showed anti-inflammatory activity. Herein, the isolation, structure elucidation, and anti-inflammatory evaluations of compounds **1**–**6** (Figure 1) are described.

## 2. Results and Discussion

### 2.1. Structural Determination

**Figure 2:**
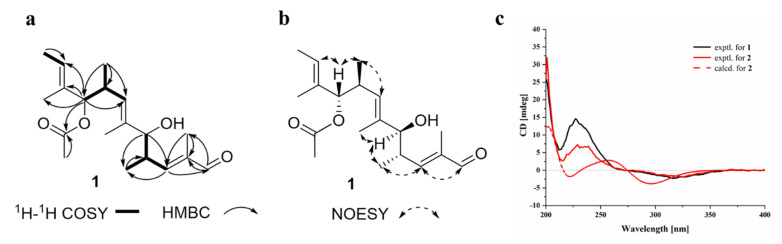
Structural assignments of **1**. Key HMBC and ^1^H-^1^H COSY correlations of **1** (**a**). Key NOESY correlations of **1** (**b**). Experimental ECD spectra of **1** and **2** and calculational ECD spectrum of **2** (**c**).

Aspormisin A (**1**) was isolated as a colorless oil, and its molecular formula was established as C_19_H_30_O_4_ by HRESIMS ion peak at *m/z* 345.2035 [M + Na]^+^ (calcd for C_19_H_30_NaO_4_^+^, 345.2036). A detailed analysis of ^1^H NMR data (Table 1) of **1** exhibited the presence of one aldehyde proton at *δ*_H_ 9.33 (s, H-1); three olefinic protons at *δ*_H_ 6.56 (dd, *J* = 9.5, 1.0 Hz, H-3), 5.43 (q, *J* = 5.5 Hz, H-11), and 5.15 (d, *J* = 9.5 Hz, H-7); two oxygenated methines at *δ*_H_ 4.87 (d, *J* = 8.5 Hz, H-9) and 3.74 (d, *J* = 7.0 Hz, H-5); two methines at *δ*_H_ 2.77–2.82 (m, H-4) and 2.69–2.74 (m, H-8); and seven methyls at *δ*_H_ 1.88 (s, H_3_-19), 1.65 (d, *J* = 1.0 Hz, H_3_-13), 1.57 (d, *J* = 1.0 Hz, H_3_-15), 1.56 (s, H_3_-12), 1.55 (s, H_3_-17), 0.89 (d, *J* = 7.0 Hz, H_3_-14), and 0.78 (d, *J* = 7.0 Hz, H_3_-16). The ^13^C NMR data and HSQC spectrum displayed nineteen carbon signals, including one aldehyde carbon at *δ*_C_ 195.4 (C-1); an ester carbon at *δ*_C_ 169.4 (C-18); three olefinic quaternary carbons at *δ*_C_ 138.0 (C-2), 137.2 (C-6), and 132.8 (C-10); three olefinic carbons at *δ*_C_ 158.6 (C-3), 127.9 (C-7), and 123.3 (C-11); four methines (including two oxygenated) at *δ*_C_ 82.3 (C-9), 78.9 (C-5), 37.3 (C-4), and 33.7 (C-8); and seven methyls at *δ*_C_ 20.9 (C-19), 17.3 (C-16), 16.7 (C-14), 12.8 (C-15), 12.2 (C-12), 11.4 (C-17), and 9.1 (C-13). The sequential ^1^H-^1^H COSY correlations (Figure 2a) between H-4 and H-3/H-5/H_3_-14, H-8 and H-7/H-9/H_3_-16, and H-11 and H_3_-12 revealed partial structures of CH-3/CH-4(CH_3_-14)/CH-5, CH-7/CH-8(CH_3_-16)/CH-9, and C-11/CH_3_-12. The above NMR data indicated that the structural skeleton of **1** was similar to that of the co-isolated 5,9-dihydroxy-2,4,6,8,10-pentamethyldodeca-2,6,10-trienal (**2**) [16]. The main distinction was the presence of an acetate group at C-9 of **1** instead of the hydroxy group in **2**, which was supported by key HMBC correlations (Figure 2a) from C-18 to H_3_-19 and H-9. Thus, the planar structure of **1** was defined as shown in Figure 1.

The configurations of the Δ^2, 6, 10^ double bonds were all deduced as *E*, based on the NOESY correlations of H_1_-1/H_1_-3, H_1_-5/H_1_-7, and H_1_-9/H_1_-11 (Figure 2b). The relative configurations of C-4/C-5 and C-8/C-9 were analyzed by proton–proton spin-coupling constant analysis. The large coupling constants, ^3^*J*_H4-H5_ (7.0 Hz) and ^3^*J*_H8-H9_ (8.5 Hz), indicated the *threo* configurations of C-4/C-5 and C-8/C-9 [17,18]. Furthermore, the absolute configuration of the linear polyketide (**1**) was alternatively determined by comparison of the experimental ECD curves with that of compound **2** (Figure 2c). From a biosynthetic point of view, they shared the well-matched experimental ECD curves (positive Cotton effects at 200 and 225 nm, and negative Cotton effect at 325 nm). According to the configuration of the closest structure of **2**, TMC-151s (Supplementary Materials, Figure S10), which was unambiguously determined by X-ray single crystal diffraction analysis [19], the ECD calculation of (2*E*, 4*S*, 5*S*, 6*E*, 8*S*, 9*S*, 10*E*)-**2** was further subjected to verify the absolute configuration as shown in Figure 2c. The well-matched experimental and calculational ECD curves revealed the (2*E*, 4*S*, 5*S*, 6*E*, 8*S*, 9*S*, 10*E*)-configuration of **2**. Finally, the absolute configuration of **1** was determined as (2*E*, 4*S*, 5*S*, 6*E*, 8*S*, 9*S*, 10*E*)-**1**.

**Figure 3:**
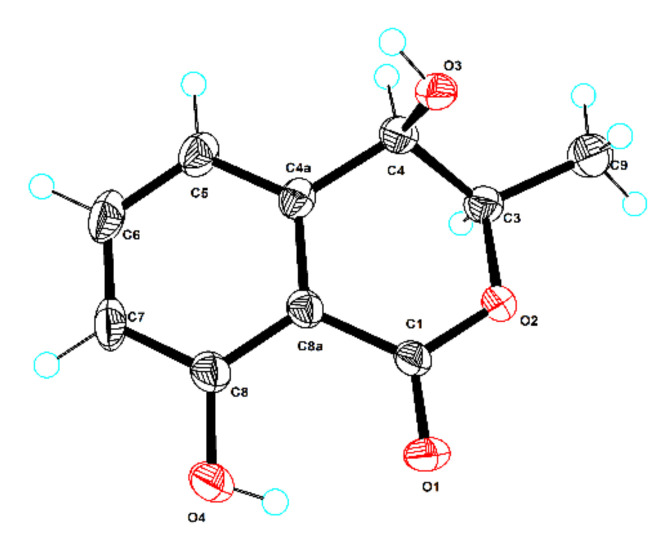
ORTEP diagram of compound **6**.

The known compounds were elucidated as 5,9-dihydroxy-2,4,6,8,10-pentamethyldodeca-2,6,10-trienal (**2**) [16], (+)-(9*R*,10*E*,12*E*)-9-methoxyoctadecadienoic acid (**3**) [20], saccharonol A (**4**) [21], (3*R*, 4*S*)-(−)-4-hydroxymellein (**5**) [22], and (3*R*, 4*R*)-(−)-4-hydroxymellein (**6**) [23], respectively, by comparing their physicochemical properties and spectroscopic data with the reported literature values. Notably, the X-ray crystal structure of **6** (CDCC 2132859, Figure 3) with a flack parameter of 0.02(6) is described herein for the first time. Linear polyketides with diverse structures are assembled by the polyketide synthase pathway. However, the minor compound **1** was likely derived from the major amount of compound **2** by a *trans*-esterification transformation during the preparation of the crude fungal extract and in the complex isolation process.

### 2.2. Compounds **2**, **5**, and **6** Inhibited the Overproduction of Nitric Oxide in a Dose-Dependent Manner

**Figure 4:**
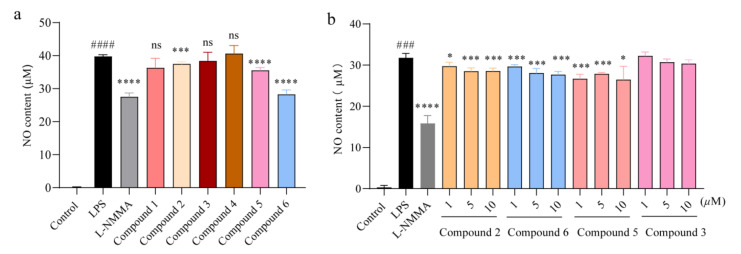
Inhibition of nitric oxide production by compounds **2, 5**, and **6.** (**a**) Cells were exposed to 0.1 μg/mL LPS and co-treated with compounds **1**–**6** (10 μM) for 24 h, respectively. NO production in the supernatant was detected after 24 h of LPS-induction, *n* = 5. (**b**) Cells were exposed to 0.1 μg/mL LPS and separately co-treated with compounds **2**, **3**, **5**, and **6** (1, 5, and 10 μM). NO production in the supernatant was detected after 24 h, *n* = 5. All data are presented as the mean ± SD of three independent experiments. *ns, p* > 0.05, * *p* < 0.05, *** *p* < 0.005, **** *p* < 0.001 vs. LPS group; ^###^ *p* < 0.005, ^####^ *p* < 0.001 vs. control group, *n* = 3. *p* value was assessed by two-tailed Student’s *t*-test.

Nitric oxide was capable of mediating inflammatory response and promoting the secretion of pro-inflammatory cytokines [24], so the production of NO in culture medium was detected after CCK-8 assay, showing that these compounds (**1**–**6**) did no damage to cell viability (Supplementary Materials, Figure S12). In brief, the systematic determination of these compounds (**1**–**6**) on the effect of NO production indicated that compound **2**, **5**, and **6** inhibited NO expression in a dose-dependent manner (Figure 4a–b). By comparison of the structural characteristics between compounds **1**, **2**, **5**, and **6**, a preliminary structure–activity relationship is discussed. The hydroxy group at C-9 in **2** played a pivotal role in NO inhibition. Moreover, the 4*R* configuration in **6** would probably increase the inflammatory activity. In general, these compounds may have the potential to fight against inflammation in view of their outstanding activity against NO accumulation. Therefore, we next evaluated the anti-inflammatory function of compounds **2**, **5**, and **6**.

### 2.3. Compounds ***2***, ***5***, and ***6*** Repressed the Secretion of Pro-Inflammatory Cytokines in Raw 264.7 Macrophages

**Figure 5:**
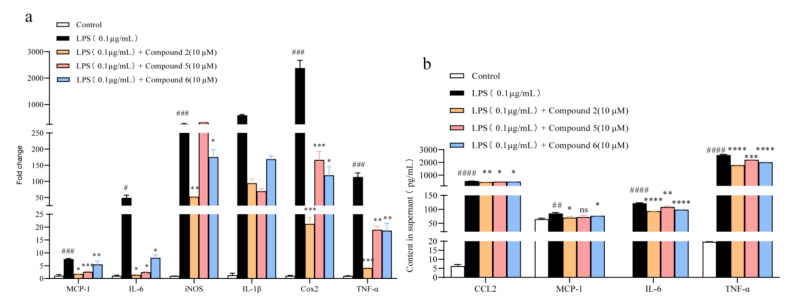
Anti-inflammation activity of compounds **2, 5,** and **6.** Cells were exposed to 0.1 μg /mL LPS and co-treated with **2**, **5**, and **6** (1, 5, 10 μM) for 24 h, respectively. (**a**) qPCR analysis of pro- inflammatory cytokines genes (MCP-1, IL-6, iNOS, IL-1*β*, Cox2, and TNF-*α*) normalized by *β*-actin, *n* = 3. (**b**) Compounds **2**, **5**, and **6** reduced the release of LPS-induced IL-6, CCL-2, TNF-*α*, and MCP-1 in culture medium, *n* = 3. All data are presented as the mean ± SD of three independent experiments. *ns, p* > 0.05, * *p* < 0.05, ** *p* < 0.01, *** *p* < 0.005, **** *p* < 0.001 vs. LPS group; ^#^ *p* < 0.05, ^##^ *p* < 0.01, ^###^ *p* < 0.005, ^####^ *p* < 0.001 vs. control group, *n* = 3. *p*-Value was assessed by two-tailed Student’s *t*-test.

A “cytokines storm” has been characterized in the inflammation response [25]; therefore, targeting the reduction in these pro-inflammatory mediators can be an effective way to block the progression of inflammation. Real-time PCR was firstly carried out to investigate the gene expression changes of these typical pro-inflammatory cytokines, such as iNOS, IL-6, IL-1*β*, TNF-*α*, Cox2, and MCP-1 (monocyte chemoattractant protein-1); it turned out that compounds **2**, **5**, and **6** could rather excellently suppress the expression of these cytokines in macrophages (Figure 5a). Further studies revealed that compounds **2**, **5**, and **6** inhibited the levels of IL-6, TNF-*α*, and MCP-1 upregulated by LPS administration in culture medium (Figure 5b). Moreover, considering that compound **2** displayed the strongest activity, we next evaluated the anti-inflammatory activity of **2** emphatically in vivo.

### 2.4. Compound **2** Attenuated the Lung Injury in LPS-Treated Mice

**Figure 6:**
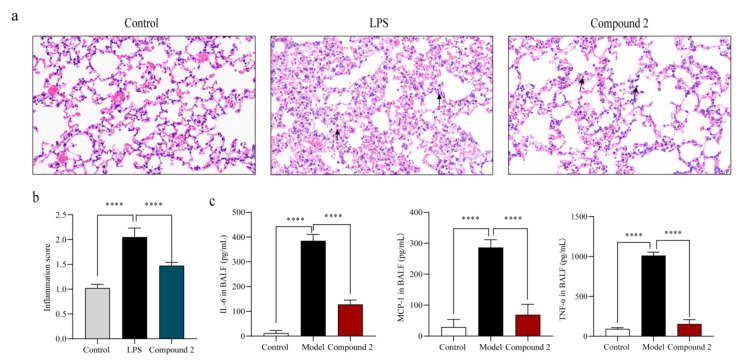
Effect of compound **2** on pathological lung injury in LPS-challenged mice. BALB/c mice were given intragastric injections of compound **2** (35 mg/kg) for 24 h before LPS administration. The mice were sacrificed after 12 h of LPS injection (5 mg/kg), and a lung histopathology was performed with hematoxylin-eosin staining (**a**). Inflammation score was measured independently by three pathologists blinded to the experiment (**b**) (*n* = 6). The levels of anti-inflammation cytokines in BALF were assayed (**c**). **** *p* < 0.001 vs. control group, *n* = 6. *p*-Value was assessed by two-tailed Student’s *t*-test.

To further verify the in vivo inhibitory effect of compound **2** on inflammation, BALB/c mice were orally administrated with **2** (35 mg/kg) for 24 h before LPS treatment (5 mg/kg). The mice were sacrificed 6 h after intratracheal injection of LPS, and their bronchoalveolar fluid (BALF) as well as ipsilateral lung lobe were collected for subsequent detection. As shown in the Figure 6a, the disordered physiological structure of the lung and the alveolar tissues were ameliorated by compound **2**. Compared with the LPS-treated group (shown with the red arrows), there was less infiltration of the inflammatory cells in the compound **2** administrated group. Besides, the cytokines level (MCP-1, IL-6, and TNF-*α*) in BALF in the compound **2** group was generally lower than that of the model group (Figure 6b). All these results suggested that compound **2** can effectively alleviate the inflammation induced by LPS in vivo.

## 3. Materials and Methods

### 3.1. General Experimental Procedures

The UV spectrum was recorded on a Shimadzu UV-2600 PC spectrometer (Shimadzu, Beijing, China). The IR spectrum was obtained using an IR Affinity-1 spectrometer (Shimadzu). Optical rotations were determined with an Anton Paar MPC 500 polarimeter (Anton, Graz, Austria). HRESIMS spectra were recorded with a Bruker maXis Q-TOF mass spectrometer (Bruker BioSpin International AG, Fällanden, Swizerland). X-ray diffraction intensity data were collected on an Agilent Xcalibur Nova single-crystal diffractometer (Santa Clara, CA, USA) using Cu Kα radiation. The NMR spectra were recorded on a Bruker Avance-500 spectrometer (Bruker BioSpin International AG, Fällanden, Switzerland), and chemical shifts were recorded as *δ*-values. Semipreparative high-performance liquid chromatography (HPLC) was performed on the Hitachi Primaide with a DAD detector, using an ODS column (YMC-pack ODS-A, 10 × 250 mm, 5 μm). Thin-layer chromatography analysis (TLC) and column chromatography (CC) were carried out on plates precoated with silica gel GF254 (10–40 μm) and over silica gel (200–300 mesh) (Qingdao Marine Chemical Factory, Qingdao, China) and Sephadex LH-20 (Amersham Biosciences, Uppsala, Sweden), respectively. Spots were detected on TLC (Qingdao Marine Chemical Factory, Qingdao Marine Chemical Factory, Qingdao, China)) under 254 nm UV light. All solvents employed were of analytical grade (Tianjin Fuyu Chemical and Industry Factory, Tianjin, China).

### 3.2. Fungal Material

The fungal strain SCSIO 41020 was isolated from the alga *Hypnea pannosa* collected from the South China Sea in Luhuitou, Sanya city, Hainan province, China (18.216° N, 109.495° E). The strain was stored on MB agar (malt extract 15 g, sea salt 10 g, agar 16 g, H_2_O 1 L, pH 7.4–7.8) slants in liquefied petrolatum and deposited at the Key Laboratory of Tropical Marine Bio-resources and Ecology, Chinese Academy of Sciences. The strain SCSIO 41020 was designated as *Aspergillus ochraceopetaliformis* due to its ITS sequence (GenBank accession No. OL884728) homology with that of *Aspergillus ochraceopetaliformis.*

### 3.3. Fermentation and Extraction

The fungal strain was cultured in 200 mL seed medium (1.5% malt extract, 0.25% sea salt pH 7.4–7.8) in 500 mL Erlenmeyer flasks at 28 °C for 3 days on a rotary shaker (180 rpm). A largescale fermentation was incubated statically at 25 °C under a normal day/night cycle for 30 days using a rice medium (80% rice, 0.25% sea salt, 0.15% bacterial peptone) in a 1 L flask (×60). The entire fermented culture was extracted with EtOAc three times to afford a rufous extract (120 g).

### 3.4. Isolation and Purification

The organic extract was subjected to silica gel CC using step gradient elution with petroleum ether/CH_2_Cl_2_ (0–100%, *v*/*v*) and CH_2_Cl_2_/CH_3_OH (0–100%, *v*/*v*) to obtain nine fractions (Frs. 1–9) based on TLC properties. Fraction 5 was subjected to semipreparative HPLC eluting with CH_3_OH/H_2_O (60–10%, 0–20 min, 2 mL/min) to afford **6** (9.2 mg, *t*_R_ = 13.5 min), **5** (23.6 mg, *t*_R_ = 15.2 min), and **4** (4.8 mg, *t*_R_ = 19.3 min). Fr. 6 was divided into five subfractions (Frs. 6-1–5) by a silica gel CC using step gradient elution with EtOAc/CH_3_OH (0–100%, *v*/*v*). Subfraction 6-2 was further purified by semipreparative HPLC (92% CH_3_CN/H_2_O, 2 mL/min) to afford **1** (3.5 mg, *t*_R_ = 9.5 min) and **3** (2.8 mg, *t*_R_ = 14.4 min). Compound **2** (58.6 mg, *t*_R_ = 24.0 min) was obtained from fraction 7 by semipreparative HPLC eluting with 52% CH_3_CN/H_2_O (2 mL/min).

### 3.5. ECD Calculation of Compound 2

Conformational analyses were carried out via Monte Carlo searching by means of the Spartan’14 software using a Molecular Merck force field. The results showed the ten lowest energy conformers within an energy window of 14 Kcal/mol. Then, these conformers were further re-optimized by TD-DFT method at the B3LYP/6-31G(d) level in methanol using the Gaussian 16 program [26]. ECD calculations were further carried out at the B3LYP/6-311+G (d, p) level in methanol by adopting 50 excited states. The ECD spectra were generated based on Boltzmann distribution theory by the SpecDis 3.0 under a half band width of 0.3 eV and shifted by −25 nm to facilitate comparison to the experimental data.

### 3.6. Spectroscopic Data of Compound ***1***

Aspormisin A (**1**): colorless oil; [*α*${]}_{D}^{25}$ +23 (*c* 0.01, CH_3_OH); UV (CH_3_OH) *λ*_max_ (log *ε*) 200 (4.27), 211 (4.00), 230 (4.23) nm; IR (film) *ν*_max_ 3446, 2964, 2927, 2258, 1732, 1683, 1240, 1205, 1138, 1021 cm^−1^; ^1^H and ^13^C NMR data, Table 1; HRESIMS *m/z* 345.2035 [M + Na]^+^ (calcd for C_19_H_30_NaO_4_^+^, 345.2036), 667.4172 [2M + Na]^+^ (calcd for C_38_H_60_NaO_8_^+^, 667.4180).

### 3.7. X-ray Crystallographic Analysis

The clear light colorless crystal of **6** was obtained in MeOH by slow evaporation. Crystallographic data for the structure have been deposited in the Cambridge Crystallographic Data Centre. Copies of the data can be obtained, free of charge, on application to CCDC, 12 Union Road, Cambridge CB21EZ, UK (fax: +44(0)-1223-336033 or e-mail: deposit@ccdc.cam.ac.uk).

Crystal data for **6**: C_10_H_10_O_4_, *M*r = 194.18, crystal size 0.16 × 0.14 × 0.10 mm^3^, monoclinic, a = 6.5123 (10) Å, b = 24.5467 (3) Å, c = 16.8673 (2) Å, *α* = 90°, *β* = 91.0470 (10)°, *γ* = 90°, *V* = 2695.88 (6) Å3, *Z* = 12, *T* = 100.00 (10) K, space group *P*2_1_, *μ*(Cu K*α*) = 0.944 mm^−1^, *D_calc_* = 1.435 g/cm^3^, 27695 reflections measured (5.24° ≤ 2Θ ≤ 148.846°), 10555 unique (*R*_int_ = 0.0315, *R*_sigma_ = 0.0356). The final *R*_1_ values were 0.0503 (I > 2*σ*(I)). The final *wR*(F^2^) values were 0.1342 (I > 2*σ*(I)). The final *R*_1_ values were 0.0538 (all data). The final *wR*(F^2^) values were 0.1366 (all data). The goodness of fit on F^2^ was 1.034. The flack parameter was 0.02(6). (CCDC 2132859).

### 3.8. LPS-Induced RAW264.7 Macrophages Inflammation Model

RAW264.7 cells, which originated from the American Type Culture Collection (ATCC) (Manassas, VA, USA), were obtained from the Peking Union Medical College. Cells were cultured in Dulbecco’s modified Eagle’s medium (DMEM) with high glucose (Corning, Corning, NY, USA) supplemented with 10% (*v*/*v*) fetal bovine serum (FBS, Gibco, Grand Island, NY, USA), penicillin (100 U/mL), and streptomycin (100 mg/mL) (Thermo Scientific, Waltham, MA, USA) with 5% CO_2_ at 37 °C. For LPS induction, RAW264.7 cells were seeded in a 24-well plate at a density of 1 × 10^5^ cells/well, after adhesion cells were exposed to 0.1 μg/mL LPS (#L2630, Merck, Shanghai, China) and separately co-treated with the compounds (at the final concentration of 10 μM) for another 24 h. Culture supernatant was collected to measure the content of NO, IL-6, TNF-*α*, and MCP-1, and cells were harvested for RNA extraction.

### 3.9. CCK-8 Assay

An amount of 3 × 10^4^ cells/well (100 μL medium/well) were seeded in a 96-well plate until adhesion; then, they were exposed to the compounds at a final concentration of 10 μM for 24 h, respectively. Whereafter, CCK-8 solution (#C0037, Beyotime Institute of Biotechnology, Shanghai, China) was added following the manufacturer’s instructions and incubated at 37 °C for 1 h. The absorbance was read at 450 nm with a microplate reader (Thermo, Waltham, MA, USA). Cell viability was calculated by (experimental group absorbance value/control group absorbance value) × 100%.

### 3.10. Nitric Oxide Production Assay

NO production was assessed with Nitric Oxide Assay Kit (#S0021, Beyotime Institute of Biotechnology, Shanghai, China) according to the manufacturer’s instructions. Briefly, after 24 h of LPS treatment, the culture supernatant (50 μL) was transferred to another 96-well plate, and Griess reagents I (50 μL) as well as Griess reagents II (50 μL) were successively added. Absorbance was read at 540 nm.

### 3.11. Cytokines Detection

Mice enzyme-linked immunosorbent assay (ELISA) kits were used to measure the contents of TNF-*α* (#PT512, Beyotime Institute of Biotechnology, Shanghai, China), IL-6 (#PI326, Beyotime Institute of Biotechnology, Shanghai, China), and MCP-1 (#PC125, Beyotime Institute of Biotechnology, Shanghai, China) in the culture supernatant collected above, according to the manufacturer’s instructions. Absorbance was measured at 450 nm.

### 3.12. Gene Expression Analysis

Total RNA was extracted from cells with TRIzol^®^ Reagent (Ambion, Austin, Texas, USA) and 1 μg total RNA was reverse transcribed into cDNA using EasyScript^®^ One-Step gDNA Removal and cDNA Synthesis SuperMix (TransGen Biotech, Beijing, China). Gene transcript levels of IL-6, TNF- *α*, MCP-1, iNOS, Cox2, and MCP-1 were quantified using the TransStart Top Green qPCR SuperMix (TransGen Biotech, Beijing, China) according to the manufacturer’s protocol. *β*-actin was used as the housekeeper gene. Primers for quantitative PCR are shown in Supplementary Materials, Table S3.

### 3.13. Animal Experimental Protocol

All animal experiments have been approved by the Medical Ethics Committee of Peking Union Medical College (approval code: SLXD—20211123056, date: 13 December 2021) and comply with the regulations of the National Institutes of Health regarding the care and use of animals in research. Briefly, 6-week-old male BALB/c mice were purchased from Vital River Laboratories Co., Ltd. (Beijing, China). The animals were randomly divided into 3 groups (*n* = 6): the control group, the model group, and the compound **2** group. Mice in the compound **2** group were orally administrated with compound **2** (35 mg/kg) for 24 h before LPS (5 mg/kg) treatment, with saline used as the control in the other two groups. After 6 h of LPS administration, the animals were sacrificed for the collection of bronchoalveolar lavage fluid (BALF) and lung tissues. The content of inflammatory cytokines in BALF was determined by ELISA, and the upper left lungs from each group (*n* = 6 per group) were fixated with 4% buffered formalin solution. Serial sections 3 μm thick were stained with hematoxylin and eosin (H&E).

### 3.14. Statistical Analysis

For statistical analysis, independent Student’s *t*-tests were used to compare the means of numerical variables. Data are presented as the mean ± SD. Statistical significance was defined as *p* values < 0.05.

## 4. Conclusions

In conclusion, six polyketides, including a new one (**1**), were isolated from the alga-derived fungus *A. ochraceopetaliformis* SCSIO 41020. The X-ray single-crystal diffraction analysis of compound **6** was reported for the first time. Compounds **2**, **5**, and **6** dose-dependently inhibited the excessive production of NO and pro-inflammatory cytokines in LPS-treated RAW 264.7 macrophages without any evidence of cytotoxicity. The preliminary structure–activity relationship is also discussed. Moreover, the greatest potential inhibitor (**2**) further attenuated the lung injury in LPS-treated mice by downregulating the levels of pro-inflammatory cytokines, including IL-6, MCP-1, and TNF-α. Our findings provide a basis for the further development of linear polyketides as potential anti-inflammatory agents.

## Figures and Tables

**Table 1:** The NMR data of compound **1** (500 and 125 MHz, *δ* in ppm, DMSO-*d*_6_).

Pos.	*δ*_C_ Type	*δ*_H_ (*J* in Hz)	HMBC	^1^H-^1^H COSY
1	195.4, CH	9.33 (s)	C-2, C-13	
2	138.0, C			
3	158.6, CH	6.56 (dd, 9.5, 1.0)	C-1, C-13	H-4
4	37.3, CH	2.77–2.82 (m)	C-3, C-14	H-3, H-5, H_3_-14
5	78.9, CH	3.74 (d, 7.0)	C-3, C-4, C-6, C-7, C-14, C-15	H-4
6	137.2, C			
7	127.9, CH	5.15 (d, 9.5)	C-5, C-15	H-8
8	33.7, CH	2.69–2.74 (m)	2.69–2.74 (m)	2.69–2.74 (m)	C-7, C-9, C-16	H-7, H-9, H_3_-16
9	82.3, CH	4.87 (d, 8.5)	C-7, C-8, C-10, C-11, C-17, C-18	H-8
10	132.8, C			
11	123.3, CH	5.43 (q, 5.5)	C-9, C-12	H_3_-12
12	12.2, CH_3_	1.56 (s)	C-11	H-11
13	9.1, CH_3_	1.65 (d, 1.0)	C-1, C-3, C-4	
14	16.7, CH_3_	0.89 (d, 7.0)	C-3, C-4, C-5	H-4
15	12.8, CH_3_	1.57 (d, 1.0)	C-5, C-6, C-7	
16	17.3, CH_3_	0.78 (d, 7.0)	C-7, C-8, C-9	H-8
17	11.4, CH_3_	1.55 (s)	C-9	
18	169.4, C			
19	20.9, CH_3_	1.88 (s)	C-18	

## Data Availability

All data is contained within the article and supporting information.

## Supplementary Materials

The following supporting information can be downloaded at: https://www.mdpi.com/article/10.3390/md20050295/s1. The spectroscopic data of **2**–**6**; the NMR, HRESIMS, UV, and IR spectra of **1**; ITS sequence data of the strain; Effect on cell viability of compounds **1**–**6**; Primers used in qPC (PDF); X-ray crystallographic data of compound **6** (CIF).
