# Supplementary material for: Anti-Inflammatory Polyketides from an Alga-Derived Fungus Aspergillus ochraceopetaliformis SCSIO 41020
Source: Mar Drugs. 2022 Apr 27;20(5):295. doi: 10.3390/md20050295 (PMC9146786; doi:10.3390/md20050295)
Supplement: Supplementary file 1 [file marinedrugs-20-00295-s001.zip › marinedrugs-1677896-supplementary.pdf]

Supporting Information

Anti-inflammatory compounds from the alga-derived fungus *Aspergillus ochraceopetaliformis* SCSIO 41020

Chunmei Chen[1,2#], Xue Ren[3#], Huaming Tao[4], Wenteng Cai[4], Yuchi Chen[4], Xiaowei Luo[5]*, Peng Guo[3]*, and Yonghong Liu[1,2,5]*

[1] CAS Key Laboratory of Tropical Marine Bio-Resources and Ecology/Guangdong Key Laboratory of Marine Materia Medica, South China Sea Institute of Oceanology, Chinese Academy of Sciences,
Guangzhou 510301, China; chenchunmei18@mails.ucas.ac.cn
[2] University of Chinese Academy of Sciences, Beijing 100049, China
[3] Capital Institute of Pediatrics, Beijing 100020, China; rlxue0529@163.com
[4] Guangdong Provincial Key Laboratory of Chinese Medicine Pharmaceutics, School of Traditional Chinese Medicine, Southern Medical University, Guangzhou 510515, China; taohm@smu.edu.cn (H.T.);
cwt0825@163.com (W.C.); cyc11136456@163.com (Y.C.)
[5] Institute of Marine Drugs, Guangxi University of Chinese Medicine, Nanning 530200, China
* Correspondence: luoxiaowei1991@126.com (X.L.); guopeng_chcip@163.com (P.G.); yonghongliu@scsio.ac.cn (Y.L.)
† These authors contributed equally to this work.

Table of Contents

The physicochemical data of the known compounds 2–6 3

Figure S1. ^{1}H NMR spectrum of aspormisin A (**1**) in DMSO-d_6. 4

Figure S2. ^{13}C NMR spectrum of aspormisin A (**1**) in DMSO-d_6. 4

Figure S3. HSQC spectrum of aspormisin A (**1**) in DMSO-d_6. 5

Figure S4. HMBC spectrum of aspormisin A (**1**) in DMSO-d_6. 5

Figure S5. ^{1}H–^{1}H COSY spectrum of aspormisin A (**1**) in DMSO-d_6. 6

Figure S6. NOESY spectrum of aspormisin A (**1**). 6

Figure S7. HRESIMS spectrum of aspormisin A (**1**). 7

Figure S8. IR spectrum of aspormisin A (**1**). 7

Figure S9. UV spectrum of aspormisin A (**1**) in MeOH 8

Figure S10. The structure and ORTEP diagram of TMC-151s. 8

ECD Calculation Details for 2. 9

Table S1. Energies of **2** at MMFF94 force field. 9

Table S2. Energies of **2** at B3LYP/6–31+g(d) level in methanol. 9

Figure S11. The The optimized conformers and equilibrium populations of **2**. 10

The strain's (*Aspergillus ochraceopetaliformis* SCSIO 41020) ITS sequence of the rDNA 11

Figure S12. Effect on cell viability of compounds 1−6 in RAW264.7 cells at the dose of 10 *μ*M. 11

Table S3. Primers used in qPCR. 11

The physicochemical data of the known compounds 2–6

5,9-Dihydroxy-2,4,6,8,10-pentamethyldodeca-2,6,10-trienal (**2**): white, amorphous solid; $[\alpha]_D^{25}$ +16 (*c* 0.01, CH_3OH); ^1H NMR (500 MHz, DMSO-d_6) δ 9.39 (1H, s, H-1), 6.58 (1H, dd, J = 9.5, 1.5 Hz, H-3), 5.42 – 5.31 (1H, m, H-11), 5.27 (1H, dd, J = 9.0, 1.5 Hz, H-7), 3.77 (1H, d, J = 7.5 Hz, H-5), 3.60 (1H, d, J = 7.5 Hz, H-9), 2.79 (1H, qd, J = 7.0, 2.5 Hz, H-4), 1.68 (3H, d, J = 1.0 Hz, H_3-13), 1.58–1.55 (6H, m, H_3-12, H_3-15), 1.53 (3H, t, J = 1.0 Hz, H_3-17), 0.87 (3H, d, J = 7.0 Hz, H_3-14), 0.76 (3H, d, J = 6.9 Hz, H_3-16). ^{13}C NMR (125 MHz, DMSO-d_6) δ 195.4 (C-1), 159.2 (C-3), 137.9 (C-2), 137.5 (C-10), 135.6 (C-6), 130.7 (C-7), 119.6 (C-11), 80.6 (C-9), 80.1 (C-5), 37.3 (C-4), 35.7 (C-8), 17.7 (C-16), 16.5 (C-14), 12.8 (C-17), 11.6 (C-15), 11.2 (C-12), 9.2 (C-13).

(+)-(9*R*,10*E*,12*E*)-9-Methoxyoctadecadienoic acid (**3**): white, amorphous solid; $[\alpha]_D^{25}$ +20 (*c* 0.01, CH_3OH); ^1H NMR (500 MHz, DMSO-d_6) δ 12.00 (1H, brs, COOH), 6.16 (1H, dd, J = 15.0, 10.5 Hz, H-11), 6.06 (1H, dd, J = 15.0, 10.5 Hz, H-12), 5.72 (1H, dt, J = 15.0, 7.0 Hz, H-13), 5.39 (1H, dd, J = 15.0, 8.0 Hz, H-10), 3.53 (1H, q, J = 7.0 Hz, H-9), 3.14 (3H, s, H_3-19), 2.20 (2H, td, J = 7.5, 3.0 Hz, H_2-2), 2.06 (2H, m, H-14), 1.50 (3H, m, H-3, 8a), 1.36 (1H, t, J = 6.5 Hz, H-8b), 1.26 (14H, m, H_2-4, 5, 6, 7, 15, 16, 17), 0.87 (3H, td, J = 7.0, 3.5 Hz, H-18); ^{13}C NMR (125 MHz, DMSO-d_6) δ 174.5 (C-1), 134.7 (C-13) , 132.4 (C-11), 131.7 (C-10), 129.5 (C-12), 81.2 (C-9), 55.4 (C-19), 35.1 (C-8), 33.7 (C-2), 32.0 (C-14), 31.2 (C-17), 29.0 (C-15), 28.6 (C-7, 6), 28.5 (C-5, 4), 24.5 (C-3), 22.1 (C-16), 13.9 (C-18).

Saccharonol A (**4**): yellow oil; ^1H NMR (500 MHz, Methanol-d_4) δ 6.31 (1H, s, H-4), 6.30 (1H, d, J = 2.0 Hz,H-5), 6.28 (1H, d, J = 2.0 Hz, H-7), 2.23 (3H, s, H_3-9); ^{13}C NMR (125 MHz, Methanol-d_4) δ 167.8 (C-6), 167.3 (C-1), 164.9 (C-8), 155.5 (C-3), 141.5 (C-4a), 105.5 (C-5), 103.4 (C-7), 102.4 (C-4), 99.5 (C-8a), 19.2 (C-9).

(3*R*, 4*S*)-(−)-4-Hydroxymellein (**5**): colorless crystal; $[\alpha]_D^{25}$ −15 (*c* 0.01, CH_3OH); ^1H NMR (500 MHz, Methanol-d_4) δ 7.56 (1H, dd, J = 8.5, 7.5 Hz, H-6), 7.07 (1H, d, J = 7.5 Hz, H-7), 6.93 (1H, dd, J = 8.5, 1.0 Hz, H-5), 4.56 (2H, m, H-3, 4), 1.47 (3H, d, J = 6.0 Hz, H_3-9); ^{13}C NMR (125 MHz, Methanol-d_4) δ 170.2 (C-1), 162.9 (C-8), 144.1 (C-4a), 137.8 (C-6), 117.7 (C-5), 117.7 (C-7), 108.0 (C-8a), 81.6 (C-3), 69.5 (C-4), 18.2 (C-9).

(3*R*, 4*R*)-(−)-4-Hydroxymellein (**6**) : colorless crystal; $[\alpha]_D^{25}$ −27 (*c* 0.01, CH_3OH); ^1H NMR (500 MHz, Methanol-d_4) δ 7.55 (1H, dd, J = 8.5, 75 Hz, H-6), 6.98 (1H, d, J = 1.1 Hz, H-7), 6.96 (1H, s, H-5), 4.72 (1H, qd, J = 6.5, 2.0 Hz, H-3), 4.55 (1H, d, J = 2.0 Hz, H-4), 1.52 (3H, d, J = 6.5 Hz, H-9); ^{13}C NMR (125 MHz, Methanol-d_4) δ 171.0 (C-1), 162.9 (C-8), 143.1 (C-4a), 137.7 (C-6), 119.8 (C-5), 118.4 (C-7), 108.4 (C-8a), 80.0 (C-3), 67.6 (C-4), 16.3 (C-9).

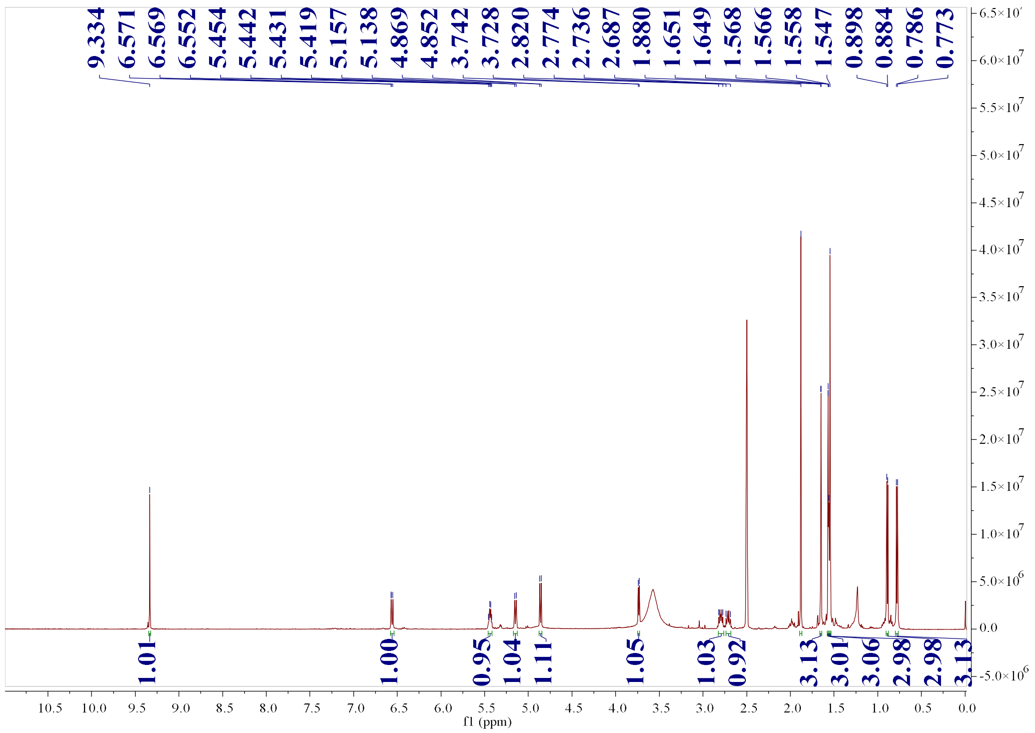

Figure S1. ^{1}H NMR spectrum of aspormisin A (**1**) in DMSO-d_6.

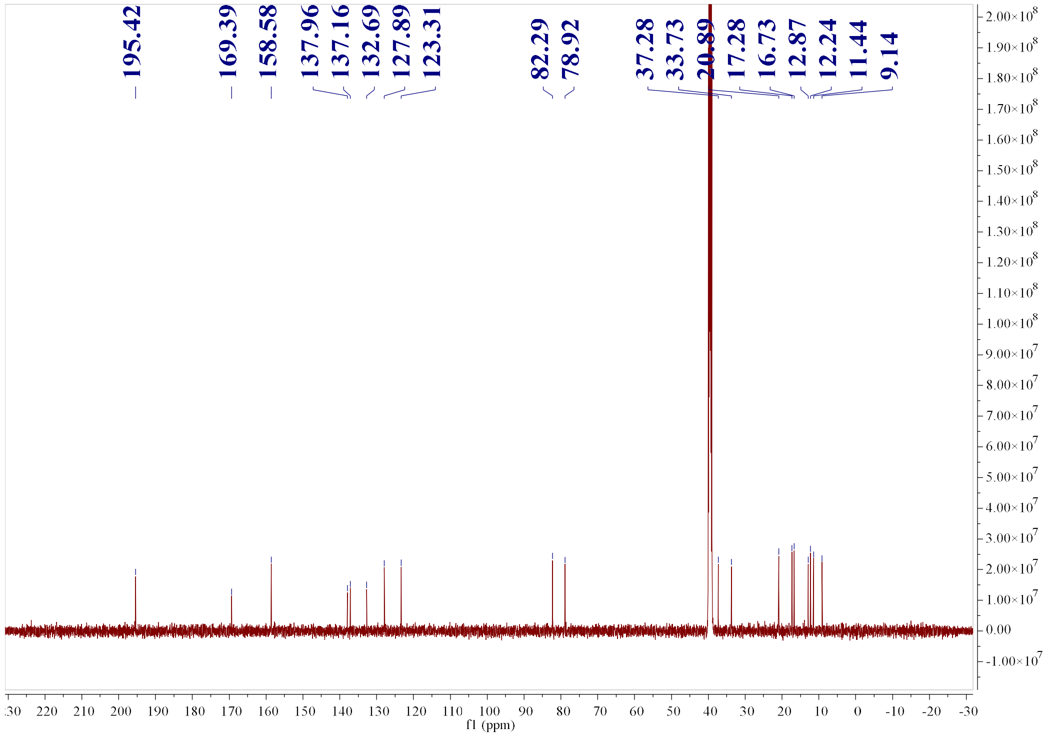

Figure S2. ^{13}C NMR spectrum of aspormisin A (**1**) in DMSO-d_6.

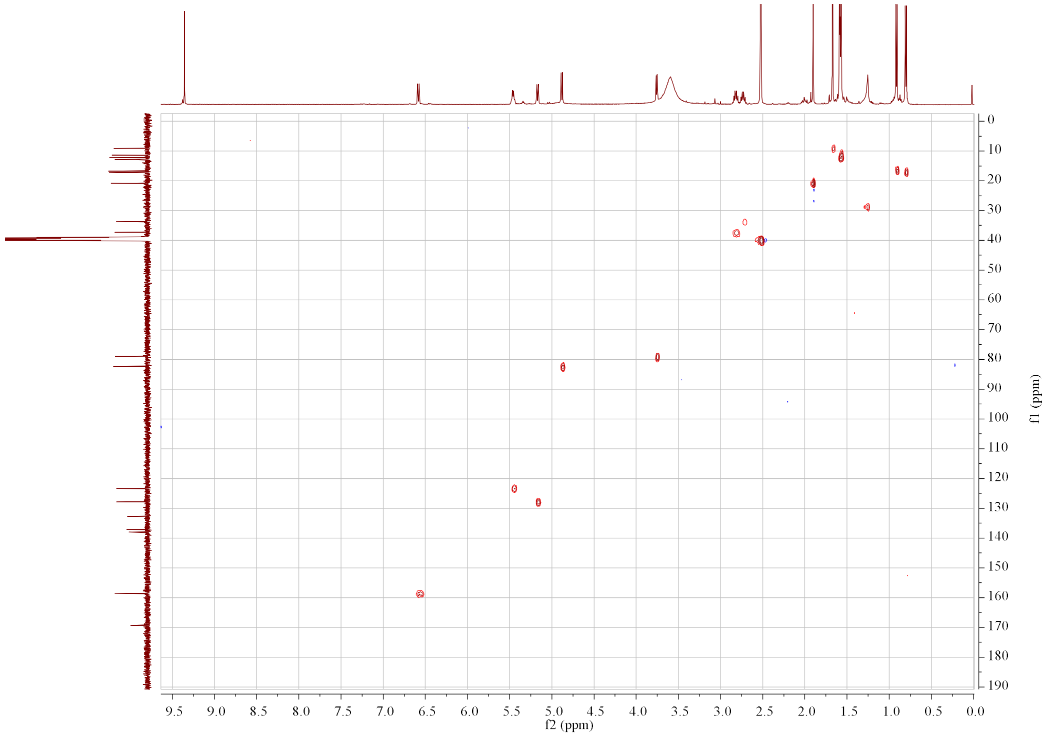

Figure S3. HSQC spectrum of aspormisin A (**1**) in DMSO-d_6.

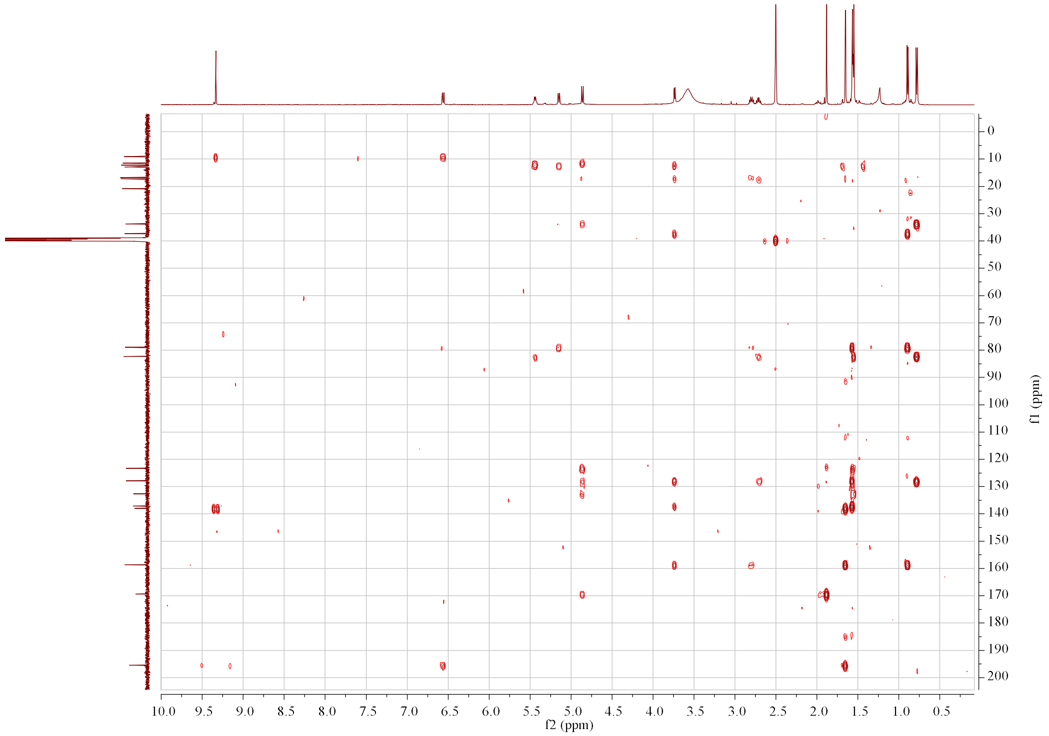

Figure S4. HMBC spectrum of aspormisin A (**1**) in DMSO-d_6.

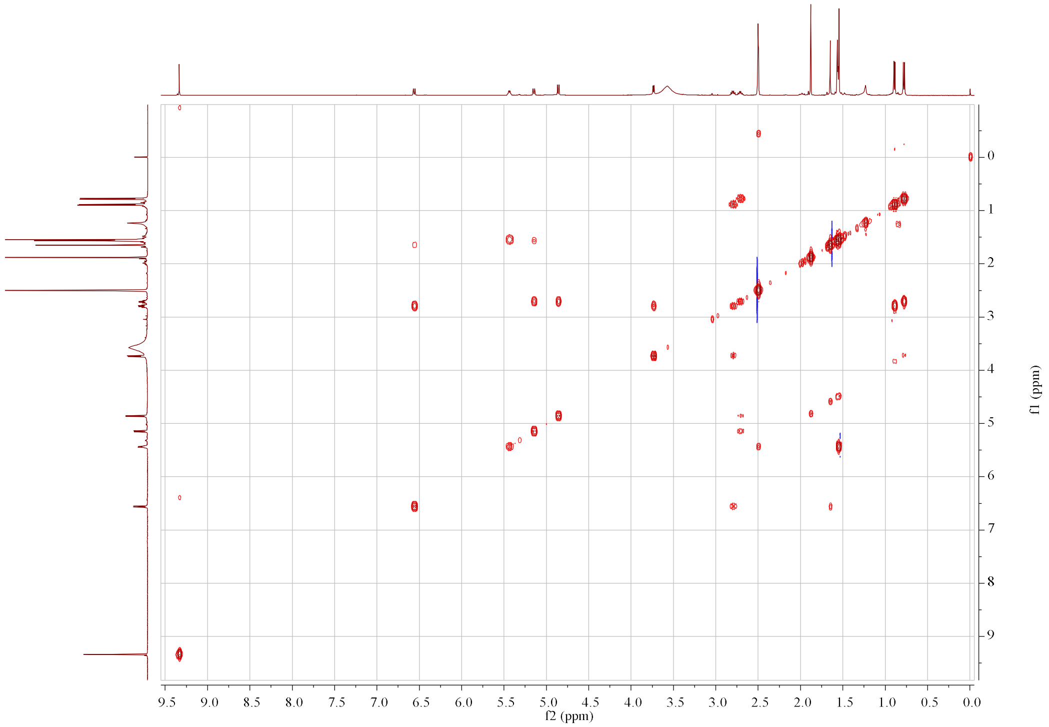

Figure S5. ^{1}H–^{1}H COSY spectrum of aspormisin A (**1**) in DMSO-d_6.

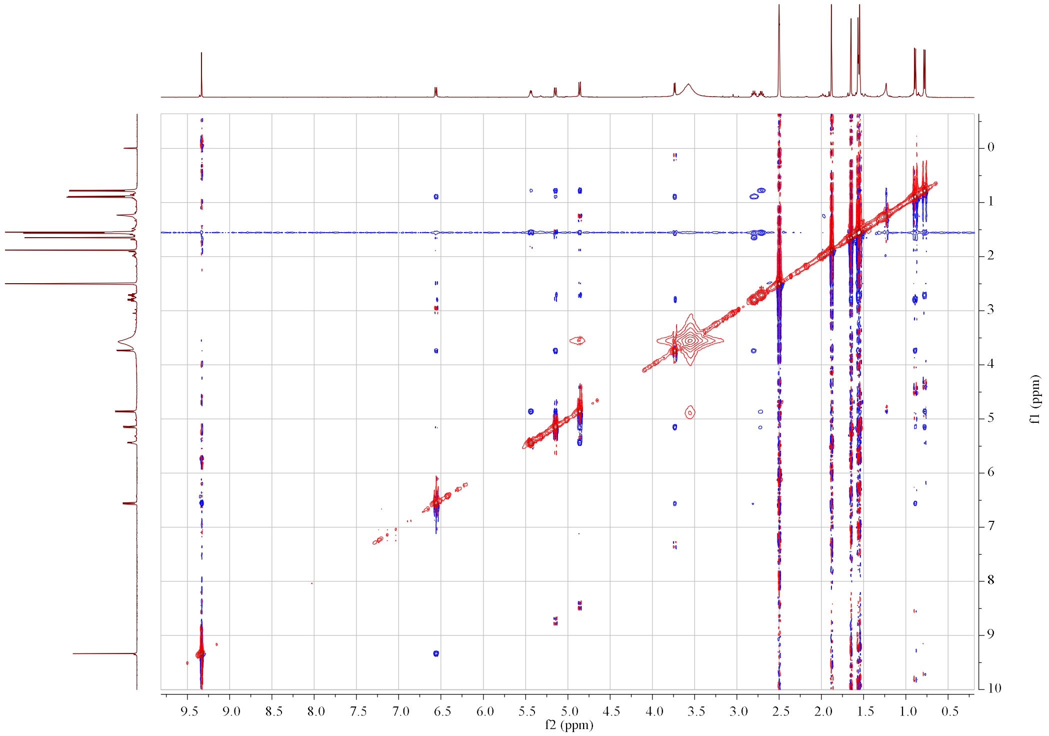

Figure S6. NOESY spectrum of aspormisin A (**1**).

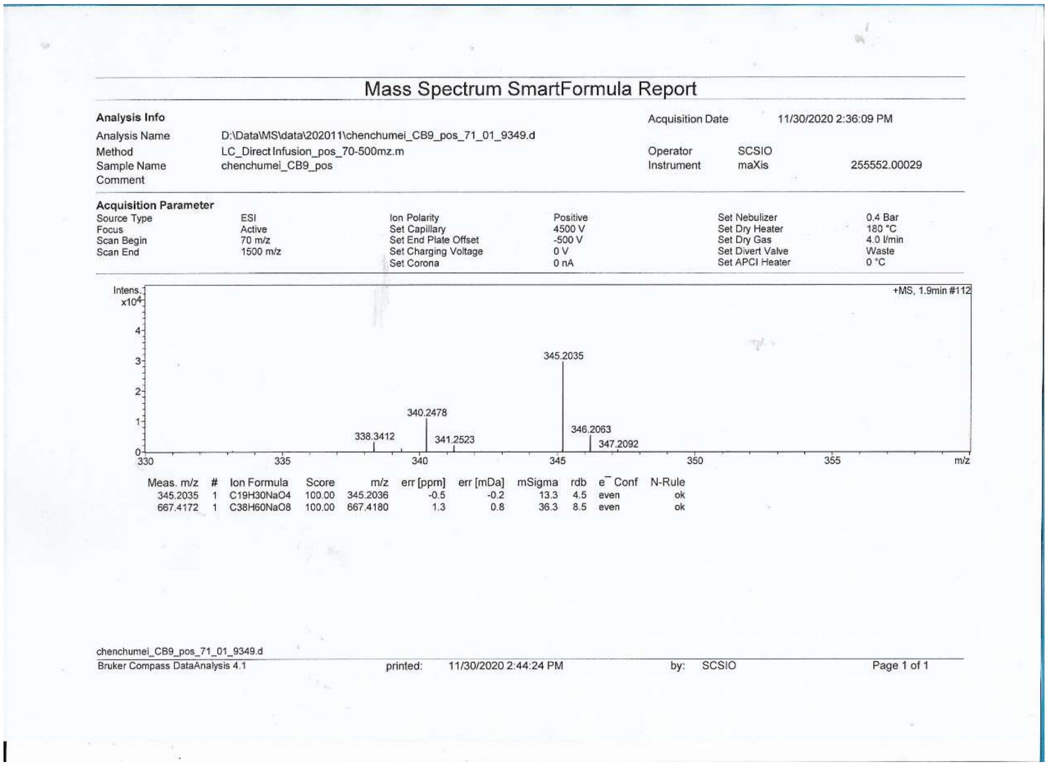

Figure S7. HRESIMS spectrum of aspormisin A (**1**).

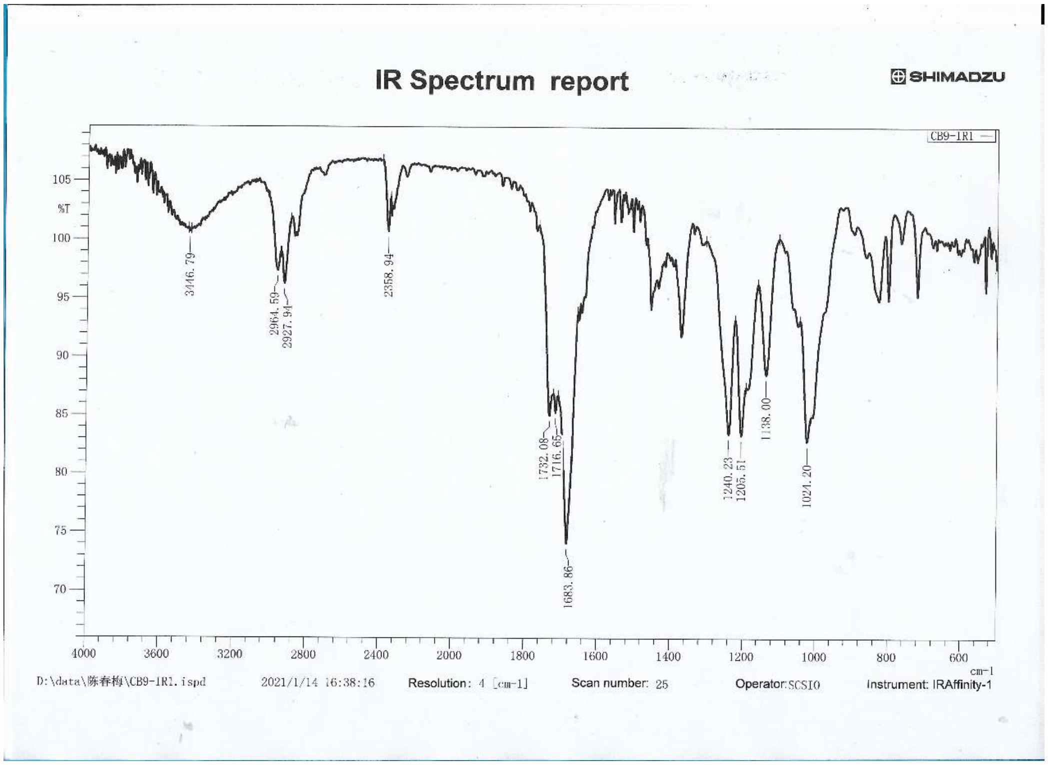

Figure S8. IR spectrum of aspormisin A (**1**).

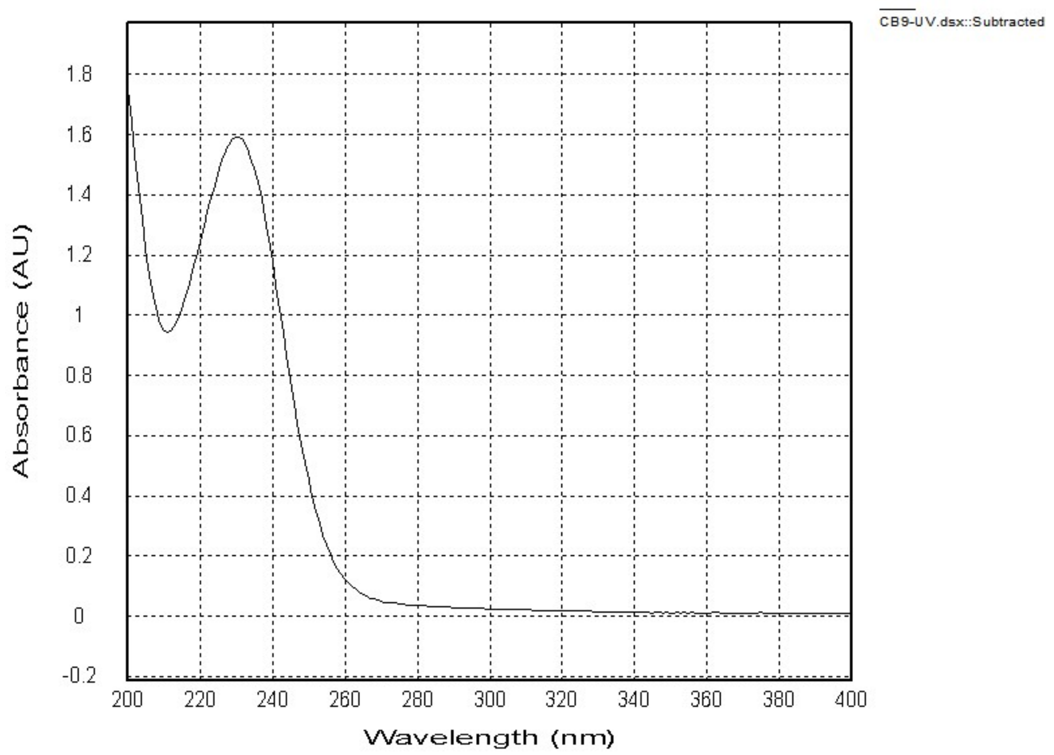

Figure S9. UV spectrum of aspormisin A (**1**) in MeOH.

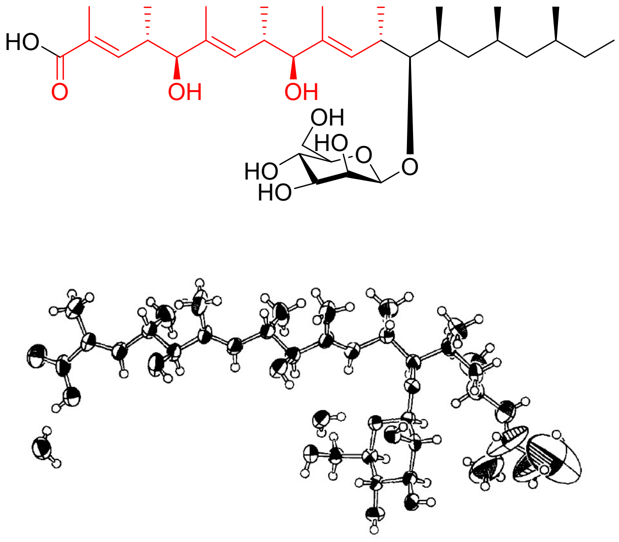

Figure S10. The structure and ORTEP diagram of TMC-151s. (Tetrahedron 1999, 55, 7771-7786.)

ECD Calculation Details for 2.

Table S1. Energies of **2** at MMFF94 force field.

Configuration	Conformer	Energy (kcal/mol)	Population (%)
**2**	1	225.35	42.4
**2**	2	225.44	40.9
**2**	3	229.58	7.7
**2**	4	230.56	5.2
**2**	5	234.09	1.2
**2**	6	235.65	0.7
**2**	7	237.29	0.3
**2**	8	237.46	0.3
**2**	9	237.73	0.3
**2**	10	239.38	0.1

Table S2. Energies of **2** at B3LYP/6–31+g(d) level in methanol.

Configuration	Conformer	E (Hartree)	E (kcal/mol)	Population (%)
**2**	1	–890.5628954	–558837.122492454	7.04
**2**	2	–890.559812	–558835.18762812	0.27
**2**	3	–890.5592548	–558834.837979548	0.15
**2**	4	–890.5599427	–558835.269643677	0.31
**2**	5	–890.5620908	–558836.617597908	3.00
**2**	6	–890.5652041	–558838.571224791	81.41
**2**	7	–890.5586898	–558834.483436398	0.07
**2**	8	–890.5627066	–558837.004018566	5.77
**2**	9	–890.5616937	–558836.368413687	1.97
**2**	10	–890.5566407	–558833.197605657	0.01

2

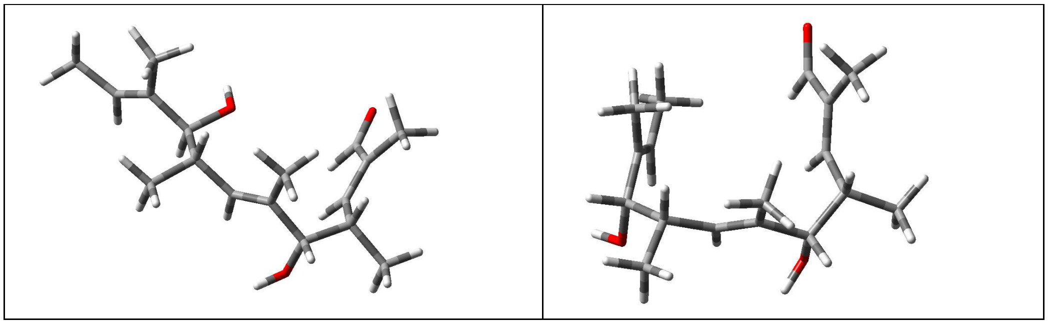

Conf.1 (7.04%)	Conf.2 (0.27%)
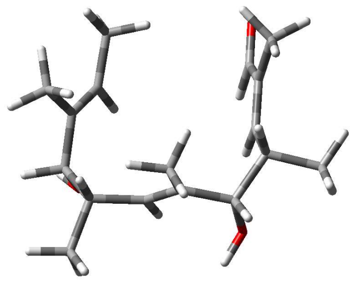	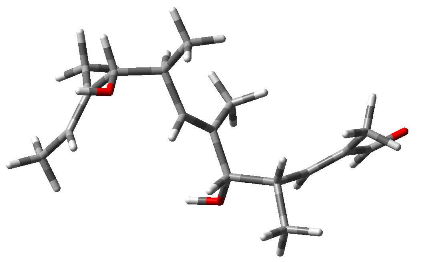
Conf.3 (0.15%)	Conf.4 (0.31%)
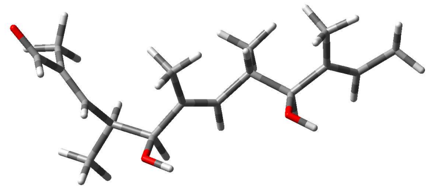	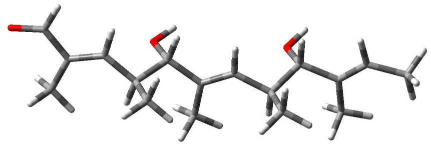
Conf.5 (3.00%)	Conf.6 (81.41%)
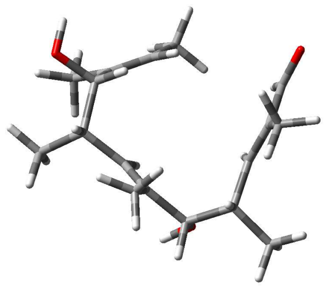	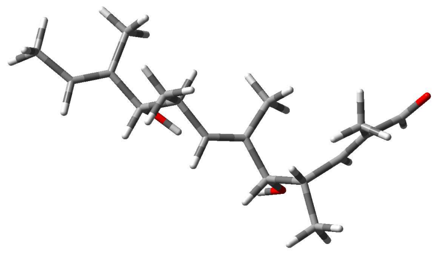
Conf.7 (0.07%)	Conf.8 (5.77%)
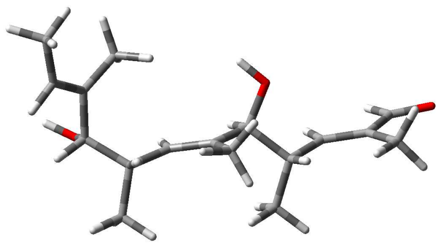	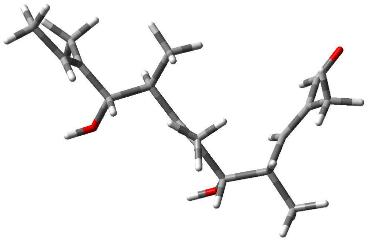
Conf.9 (1.97%)	Conf.10 (0.01%)

Figure S11. The The optimized conformers and equilibrium populations of **2**.

The strain's (*Aspergillus ochraceopetaliformis* SCSIO 41020) ITS sequence of the rDNA

TCCTCCGCTTATTGATATGCTTAAGTTCAGCGGGTATCCCTACcTGATCGAGGTCACCTGG AGAATAATGGTTGCTTTTCAGCGTCGGCCAGCGCCGGCCGGGCCTACGAGAGCGGTGT GACAAAGCCCCATACGCTCGAGGACCGGACGCGGTGCCGCCGCTGCCTTTCGGGCCCG TCCCCCGGGGGGGACGAGGACCCAACACACAAGCCGGGCTTGAGGGCAGCAATGACG CTCGGACAGGCATACCCCCCGGAATACCAGGGGGTGCAATGTGCGTTCAAAGACTCGAT GATTCACTGAATTCTGCAATTCACATTAATTATCGCATTTCGCTGCGTTCTTCATCGATGC CGGAACCAAGAGATCCATTGTTGAAAGTTTTAACTGATTGCGATACAATCGAACTCAGA CGACAAAACTTCAGACAGTGTTCACGTTGGGGTCTCCGGCGGGCGCTCGCCCGGGGGG AGGGGTTCCCCCCCGGCGGCCGCGCGAACGGCGGGCCCGCCGAAGCAACTTGGTACA GTATACAAGGGTGGGAGGTtGGGCCCCGAAGGAACCCTCACTCAGTAATGATCCTTCCG CAGGTTCACCTACGGAAG

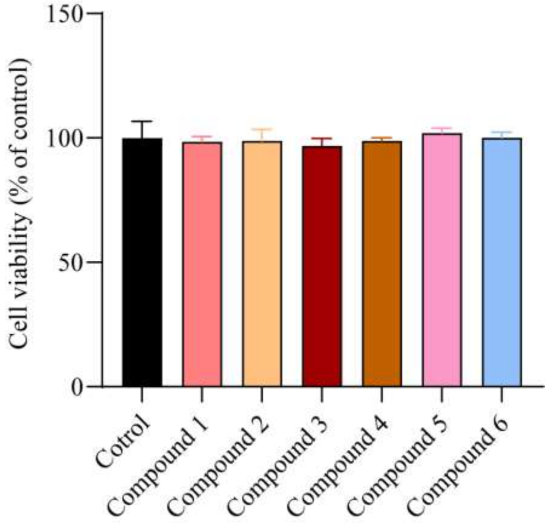

Figure S12. Effect on cell viability of compounds 1−6 in RAW264.7 cells at the dose of 10 μM. Cells were exposed to compounds **1− 6** (10 μM) for 24 h, respectively. All data are presented as the mean ± SD of three independent experiments, n=6.

Table S3. Primers used in qPCR.

Name	Species	Forward (5'-3')	Reverse (5'-3')
IL-6	Mice	CCGGAGAGGAGACTTCACAG	TGGTCTTGGTCCTTAGCCAC
iNOS	Mice	CCTTACGAGGCGAAGAAGGACAG	CAGTTTGAGAGAGGAGGCTCCG
Tnf-*a*	Mice	GACCCTCACACTCAGATCAT	TTGAAGAGAACCTGGGAGTA
Cox2	Mice	CATCCCCTTCCTGCGAAGTT	CATGGGAGTTGGGCAGTCAT
IL-1β	Mice	TTCCCCAGGGCATGTTAAGG	GTCTTGGCCGAGGACTAAGG
Mcp-1	Mice	TTAAAAACCTGGATCGGAACCAA	GCATTAGCTTCAGATTTACGGGT
β-actin	Mice	ACACTGTGCCCATCTACGAG	CAGCACTGTGTTGGCATAGAG
